# PRECISE Scale: a quantitative classification for androgenetic alopecia and its application to hair transplantation

**DOI:** 10.1007/s00266-023-03462-4

**Published:** 2023-06-26

**Authors:** Felipe Pittella, Carlo G. Castro, Roberto Trivellini, Pradeep Sethi, Simone La Padula, Baltazar Sanabria, Camila Q. Paes, Miguel G. S. Palmieri, Frederico Pittella

**Affiliations:** 1 Pittella Day Hospital - Hair Restoration and Plastic Surgery, Vitoria, ES 29050-300 Brazil; 2 Trivellini Hair Restoration, 29003 Málaga, Spain; 3 Eugenix Hair Sciences, Mumbai, 122018 India; 4 Department of Plastic and Reconstructive Surgery, Università degli Studi di Napoli Federico II, 80131 Naples, Italy; 5 Department of Plastic, Reconstructive and Maxillo Facial Surgery, Henri Mondor Hospital, University Paris XII, 94000 Paris, France; 6 Clínica Sanabria, Campo Grande, MS 79040-830 Brazil; 7 Faculdade de Enfermagem, Universidade Federal de Juiz de Fora, Juiz de Fora, MG 36036-900 Brazil; 8 Faculdade de Farmácia, Universidade Federal de Juiz de Fora, Juiz de Fora, MG 36036-900 Brazil

**Keywords:** Alopecia, Hair, Hair follicle, Surgery, Plastic

## Abstract

**Background:**

Androgenetic alopecia (AGA) is a prevalent genetic condition that can affect both male and female, and is considered the most frequent form of hair loss. Traditional scales and methods of classifying AGA are basically qualitative.

**Objective:**

This work aims to propose a quantitative scale to classify AGA in order to assist hair transplantation surgery.

**Methods:**

Based on whole hairless and thinning areas that needs to receive follicular units in a hair transplantation procedure, basic equations to support the scale are proposed. Additionally, the study involves simulations that apply the classification system and compare its results with those of qualitative methods.

**Results:**

The PRECISE scale utilizes a range of 0–10, using 30 cm^2^ as the measured standard of a bald area. For hair transplantation, 1500 follicular units (FU) are recommended for each score in the PRECISE scale. Technological and manual methods to measure the hairless and thinning areas are presented and discussed. This new quantitative classification, combined with different and complementary methods of measurement of hairless and thinning areas endorse the understanding of the clinical condition by the patient and the planning of a surgery procedure.

**Conclusion:**

The developed PRECISE scale brings a different way of classifying Androgenetic alopecia (AGA), through an essentially quantitative evaluation. It can be used to elaborate the best strategy for the hair transplantation surgery and to improve the outcomes.

**Level of Evidence V:**

This journal requires that authors assign a level of evidence to each article. For a full description of these evidence-based medicine ratings, please refer to the Table of Contents or the online Instructions to Authors www.springer.com/00266.

## Introduction

Male-Pattern hair loss (MPHL) or Androgenetic alopecia (AGA) is an extremely common problem that afflicts men in the post pubertal period. Although the prevalence differs according to race and ethnicity, there is a universal distribution and increase with age [1].

Historically, a wide variety of classifications approach the subject in a qualitative way. One of the first classification was done by Beek (1950), who classified MPHL on frontal and fronto-vertical [2]. One year later, Hamilton (1951) focused on a more detailed proposal from the fronto-parietal and frontal recesses and on the vertex miniaturization [3]. Later, Ogata (1953) worked on subtypes contemplating patterns of the Japanese population [4]. By considering the inclusion of Afro-descendants in his studies, Setty (1970) simplified Hamilton's work from 1951 [5]. Further, Norwood (1975) refined Hamilton's classification from the observation that miniaturization begins in the temples, as well as at the crown/vertex, progressing to the top of the scalp [6]. This last one is known as the Norwood-Hamilton scale, which is considered the most used scale in the western world.

Since the scale of Norwood-Hamilton, several researchers and doctors again proposed new qualitative classifications. By introducing a simplified classification of baldness based on Caucasian Europeans, Bouhanna proposed their own scale in 1976 [7]. Blanchard and Blanchard [8] based their classification on measurements taken at six anatomical landmarks in the scalp. In the year 2000, Koo used the shape of the bald areas to develop a new categorization [9]. In the same year, Bouhanna launched a very detailed classification with parameters such as: fixed distances from the face, mobility and thickness of the scalp and hair covering power [10]. Seven years later, BASP (2007) was created as a gender independent classification and based on anterior hairline shape and frontal and vertex hair density combining basic and specific types [11]. Finally, the works done by Ludwig [12] and Savin [13] highlighted the female-pattern hair loss (FPHL).

When evaluating such classifications, we noticed limitations in terms of detailed description, practicality for clinical assessment, and reproducibility of classification [14]. Moreover, different patients with the same class of baldness on traditional scales, could obtain a poor aesthetical outcome in the post-procedure if approached with the same surgical strategy. This is because those classifications are essentially qualitative and lack features needed to make a proper strategy for the hair restoration.

Another fact to be considered is that there is a relevant level of divergence between the examiners when using those qualitative classifications. In other words, a single patient can be classified in different classes by different professionals using the same scale; and a mathematical classification could minimize such disagreements. Thus, a proper measurement of the area to be implanted can decisively influence the results of the hair transplantation (HT), a fact that may be even more relevant when considering professionals who are beginners in the hair restoration field. For the experienced surgeons, a quantitative classification may further improve their surgical planning leading to results of the utmost excellence.

The present work aims to propose a mathematical classification of AGA, through a quantitative evaluation. The PRECISE classification is based mainly on the areas that need to be restored. It brings, in a simple way, mathematics to assist the surgical planning. Ultimately, the new scale has potential to result in a precise hair restoration planning and function as an essential parameter for either in-person or telemedicine assessments.

## Materials and Methods

### PRECISE Scale (Precise Scalp Area Count Scale)

After observing the need for a more accurate surgical planning for hair transplantation, our medical group invited experienced surgeons from different countries and regions to work together on the development of a new quantitative classification for androgenetic alopecia, the PRECISE scale.

**Fig. 1:**
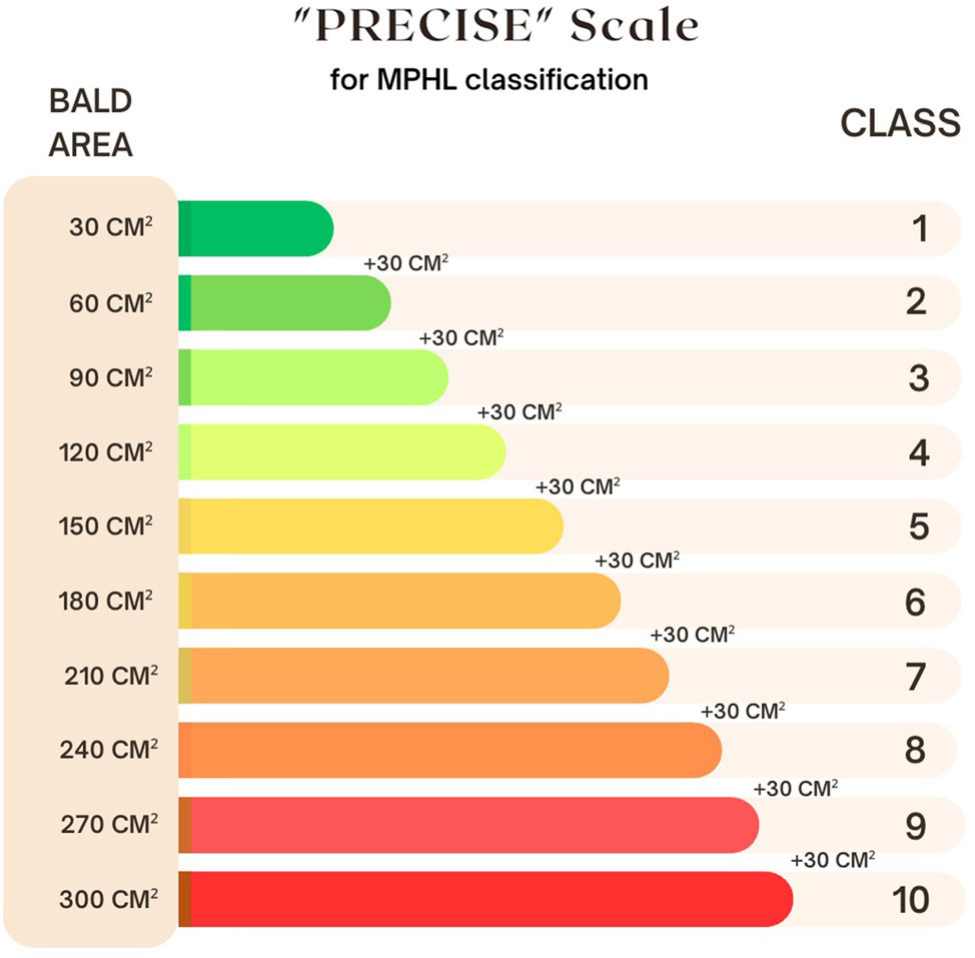
A diagram of the PRECISE Scale, for classification of AGA based on the size of the hairless and corrected thinning areas

The PRECISE classes of a MPHL range from 0 to 10 (Fig. 1). To determine it on a patient with AGA the examiner needs to measure the whole hairless and thinning areas to be restored. For every 30 cm^2^ of hairless area the patient will score 1 point in the scale. The total area to be accounted for should be the sum of the hairless and thinning androgen-dependent regions that might be approached in a hair restoration surgery.

The PRECISE class can be calculated using the Equation 1:

1 $${\text{PRECISE}}\;{\text{Classification}} = {\text{RBA/30}} + {\text{TS}}$$

where RBA = Relative Bald Area; TS = Temple Score.

The Relative Bald Area (RBA) represents the entire area with complete absence of hair added to areas of diffuse thinning that were corrected by the Hair Miniaturization Index (HMI). The calculation for correction will be presented further.

In addition, the Temple Score (TS) should be used when temporal restoration is needed. For that, the temporal recess should be classified in “Small, Medium or Large”, as follows:

- Small: each temple scores 0.1 in the PRECISE scale;
- Medium: each temple scores 0.2 in the PRECISE scale;
- Large: the androgen-dependent hair in the temporal area has miniaturized completely, thus the temporal hairline starts at the donor area above the ear. Each temple scores 0.3 in the PRECISE scale.
- If the temples are not receded, the TS should be considered ZERO.

When the examined patient has diffuse thinning regions, a correction factor should be applied to the measurement of the Relative Bald Area (RBA). The correction factor was named “Hair Miniaturization Index'' (HMI) as it determines how thinned the hair in those areas are, compared to the donor area. The equation 2 is used to correct the diffuse thinning area as described below:

2 $${\text{RBA}} = {\text{CMA}} + \left( {{\text{HMI}} \times {\text{PMA}}} \right)$$

where RBA = Relative Bald Area; CMA = Completely Miniaturized Areas; HMI = Hair Miniaturization Index; PMA = Partially Miniaturized Areas.

Completely Miniaturized Areas (CMA) refer to those areas that may need the same number of grafts as a hairless area in a hair transplantation (HT) procedure. Particularly, CMA presents HMI above 70%, and, therefore, does not need to be corrected by HMI. Partially Miniaturized Areas (PMA) are areas with HMI between 30 and 70% and must have its measurement multiplied by the HMI found so that this area can be equated to a smooth area and scored accordingly in the scale. Finally, areas with HMI below 30% do not score in the classification since they may not be addressed in a HT as they may improve with clinical treatment and no grafts may be transplanted to them.

The HMI can be determined empirically, based on a trichoscopic comparison [15] of the donor area with the evaluated area. It is also possible to determine it by comparing the moistened hairless areas with the donor area, using tactile sensitivity and adequate lighting to assess the volume of hair in the respective regions and estimate how much the hair has miniaturized.

The HMI indicates the percentage of overall loss that the hair in that territory has suffered, e.g., if a region has an HMI of 50% it means that the hair of that area has reduced 50% compared to the patient's donor area. This index considers the variation of the diameter that affects the hair of the androgen-dependent areas assessed by trichoscopy (Variability) [15], the quantitative reduction of the Hair Shafts Diameter (HSD) and the reduction of the number of hairs on those zones using the Hair Diameter Index (HDI) equation [16]. The HMI is expressed by equation 3:

3 $${\text{HMI}} = {\text{VR}} \times \left( {{\text{HDID}} - {\text{HDIR}}} \right) \div {\text{HDID}}$$

where HMI = Hair Miniaturization Index (percentage); VR = Variability; HDI = Hair Diameter Index (D = Donor area; R = Recipient area).

Variability (VR) is the percentage of hair that is different from the expected diameter of the patient's terminal hair on the assessed area and can be determined by trichoscopy [15]. A low VR means that most of the HSD of the area assessed are similar to the patient’s terminal hair diameter. A high degree of variability is found if a large percentage of the HSD are far (diverse) from the patient's terminal HSD [17].

The second part of the equation uses the HDI to show the percentage of reduction in the HSD on the assessed area. In other words, it shows how much the hair of the recipient area has thinned in comparison with the donor area. The HDI is an absolute value and can be calculated using Equation 4, where HSD is measured in microns.

4 $${\text{HDI}} = {\text{HSD}} \times \left( {{\text{hairs/cm}}^{2} } \right) \div 100$$

Moreover, for comparison purposes, the HDI can be exchanged by the Hair Coverage Value (HCV) [18] to fit the PRECISE Scale. Also, the use of decimal values in the scale is recommended for an accurate correlation with the number of grafts to be implanted. Every 3 cm^2^ of RBA scores 0.1 points.

One of the most valuable objectives of the PRECISE classification is to define surgical conduct and help the surgeon to elaborate the best strategy for the hair restoration of any patient. For that, to achieve a good standard result transplanting 1500 follicular units (FU) for each score in the PRECISE scale is recommended.

### Simulation and Comparison of the PRECISE Classification with a Traditional Scale

To provide practical examples of the PRECISE scale classification, model samples were created with the software Pixelmator Pro® (ver. 3.3) to simulate classes of baldness in different patients using Norwood-Hamilton qualitative scale. Then, for each area in every simulation, HMI, CMA, PMA and TS values have been defined. The measurement of the bald area by using these parameters allows the calculation of the PRECISE class and the follicular units recommended by the scale.

In a first simulation, two patients classified as Norwood-Hamilton 3V with different head sizes were compared, and reclassified using PRECISE scale. Further, in a second simulation, two patients classified as Norwood-Hamilton 5 (larger head size) and 6 (smaller head size) were also compared and reclassified in PRECISE scale.

## Results

Models with different degrees of baldness and characteristics were created in order to provide samples for comparison of qualitative and quantitative scales. The examples below compare how different classes would behave in the PRECISE scale.

**Fig. 2:**
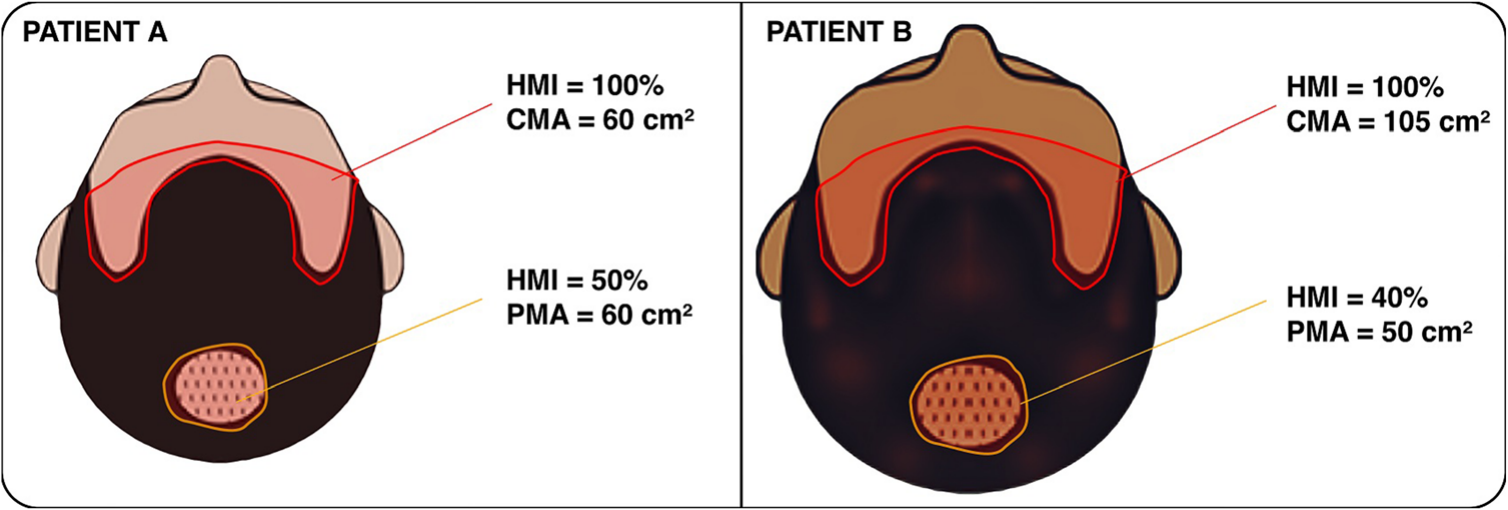
Templates showing Norwood-Hamilton class 3V with different head sizes. Temporal restoration was not necessary in those patients (TS = 0)

In this first example, 2 patients (A and B) are classified in the same Norwood-Hamilton class 3V (BASP M2V1). However, patient A has a smaller head compared to patient B (Fig. 2).

For a side-by-side comparison, Table 1 shows the measurements of the hairless area and the classification of both patients A and B according to PRECISE Scale.

**Table 1:** Comparison of area measurements of patients A and B, and the PRECISE classification

PATIENT A (Norwood-Hamilton 3V)	PATIENT B (Norwood-Hamilton 3V)
Area measured: CMA = 60 cm^2^ PMA = 40 cm^2^ (HMI 50%) TS = 0	Area measured: CMA = 105 cm^2^ PMA = 50 cm^2^ (HMI 40%) TS = 0
RBA = 60 + 40 × 0.5 = 80 cm^2^	RBA = 105 + 50 × 0.4 = 125 cm^2^
PRECISE = 80/30	PRECISE = 125/30
PRECISE = class 2.6	PRECISE = class 4.1
Recommended number of grafts: 2.6 × 1500 = 3900 grafts	Recommended number of grafts: 4.1 × 1500 = 6150 grafts

**Fig. 3:**
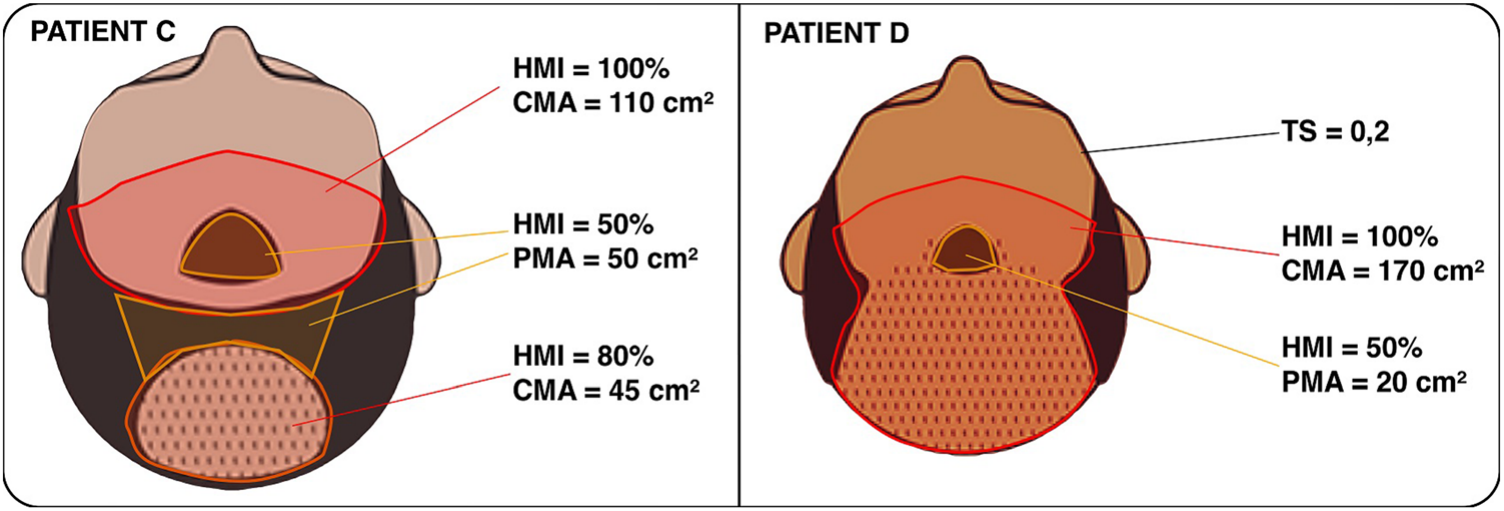
Patient C Norwood-Hamilton class 5 with a large head; Patient D Norwood-Hamilton class 6 with smaller head

In another situation, two patients (C and D) with different classes on the Norwood-Hamilton scale were compared. They were classified as class 5 and class 6 respectively in Norwood-Hamilton scale. Patient C has a larger head compared to patient D (Fig. 3).

Table 2 shows the measurements of the hairless area of patients C and D, and the classification according to PRECISE Scale.

**Table 2:** Comparison of area measurements of patients C and D, and the PRECISE classification.

PATIENT C (Norwood-Hamilton 5)	PATIENT D (Norwood-Hamilton 6)
Area measured: CMA = 155 cm^2^ PMA = 50 cm^2^ (HMI 50%) TS = 0	Area measured: CMA = 170 cm^2^ PMA = 20 cm^2^ (HMI 50%) TS = 0.2
RBA = 155 + 50 × 0.5 = 180 cm^2^	RBA = 170 + 20 × 0.5 = 180 cm^2^
PRECISE = 180/30	PRECISE = 180/30 + 0.2
PRECISE = class 6	PRECISE = class 6.2
Recommended number of grafts: 6 × 1500 = 9000 grafts	Recommended number of grafts: 6.2 × 1500 = 9300 grafts

## Discussion

One of the goals of the PRECISE scale is to estimate the number of grafts needed to obtain good coverage of the bald and thinning areas. The number of 1500 grafts for each point in the PRECISE scale is recommended. This number of grafts was calculated, as suggested by other colleagues, adopting 50 FU per cm^2^ as a reasonable filling value for most regions of the scalp, which would give a density of around 100 hairs per cm^2^ [10]. Naturally, the amount of 1500 FU for each point in the scale is only a guidance as this number may vary depending on the density that the surgeon wants to apply on the different zones of the scalp.

The choice for a 0-10 scale over the absolute values of the area is necessary for three main reasons:

- The simplicity that a 0–10 rating scale brings to the patients' understanding of the extent of their problem;
- Possibility of standardization for future research;
- Translate for the hair restoration surgeon an objective goal for surgical planning other than mere qualitative assessment.

The fact that the traditionally used scales for MPHL classification are essentially qualitative makes them unsuited for an accurate hair transplantation planning. This is because they lack features needed to make an appropriate strategy for the hair restoration and may guide the surgeon to a misjudgment on the amount of grafts he will need to transplant to achieve a proper hair restoration. This becomes evident when we consider that two patients with the same Norwood-Hamilton Class 3 may have different cranial shapes and sizes. To address these differences, two simulations were conducted for the sake of comparison.

In the example of Figure 2 and Table 1, despite both patients having the same Norwood-Hamilton class 3, patient A is classified as PRECISE class 2.6 and patient B as PRECISE class 4.1. These 2 patients with the same baldness class will need completely different amounts of grafts to achieve a good result. While for patient A the recommended number of grafts would be 3900, for patient B it would be 6150 grafts.

In a new simulation, Figure 3 and Table 2 show patients C and D with different classification of Norwood-Hamilton. Although with different classes of baldness in a qualitative scale, two patients may have suchlike PRECISE class and subsequently be approached with similar hair restoration strategy. In this case, both patients C and D may be recommended to transplant a similar number of grafts (around 9000 grafts).

Thereby, the PRECISE scale intends to quantify MPHL, assist in its progression and guide its surgical restoration. The scale presents good practicality, allowing a quick evaluation within around 5 minutes from sizing the areas to calculation. It’s basically based on the measurement of the hairless and thinning areas and applying the correction factor for the miniturized areas.

Thus, the measurement of the RBA is critical for the classification on the PRECISE scale. There are several ways to measure hairless areas. All of them have their advantages and disadvantages and may vary regarding their simplicity and precision. The exact measurement of the areas is the most valuable feature to classify MPHL properly.

For clinical purposes, the limits of the Relative Bald Area (RBA) must be clearly identified:

- Anterior limit: attachment of the frontalis muscle on the epicranial aponeurosis (galea aponeurotica): this landmark is the anterior natural limit of the hairline and is identified by asking the patient to raise the eyebrows. By doing so, he will contract the frontalis muscle and a ridge will be identifiable on the forehead. This marks the end of the muscle fibers as they attach to the galeal;
- Lateral and Posterior: These limits are set by the line surrounding the thinning zones in which the HMI is lower than 30%, i.e., the areas that are not receiving grafts in the surgery. Notice that in very advanced baldness the limit may be the patient's donor area.

The measurement of the hairless and thinning areas should start from the anterior limit and extend to the entire androgen-dependent region. For the PRECISE classification, all the regions with HMI greater than 30% must be considered. The areas of the temporal recesses are not included on this measurement as they are assessed differently.

There are several techniques to measure hairless and thinning areas, including traditional and technological methods, which are presented in the following subtopics.

### Measurement of Hairless and Thinning Areas

#### Measurement Over a 3D Model’s Mesh

A rendered 3D model from a patient’s head can be used to measure the RBA through the measurement of the 3D mesh created by LiDAR/TrueDepth or other similar technologies. The 3D mesh refers to a representation of the surface of the objects captured by scanning the environment using laser beam and/or infrared sensors and cameras.

In Light Detection and Ranging (LiDAR), a laser light is sent from a source (transmitter) and is reflected from objects in the scene. The reflected light is detected by the system receiver and the time of flight is used to develop a distance map of the objects in the scene. This technology is used, among other things, for topography, being able to map the surface of large areas and examining both natural and manmade environments. Nowadays it can also be found in various devices such as tablets and mobile phones and together with depth-sensor cameras it is made possible to map and render small objects with millimetric precision. For our purpose, LiDAR technology can measure the bald and thinning areas with high accuracy in the tree-dimensional plane despite the distortions determined by the cranial curvature.

**Fig. 4:**
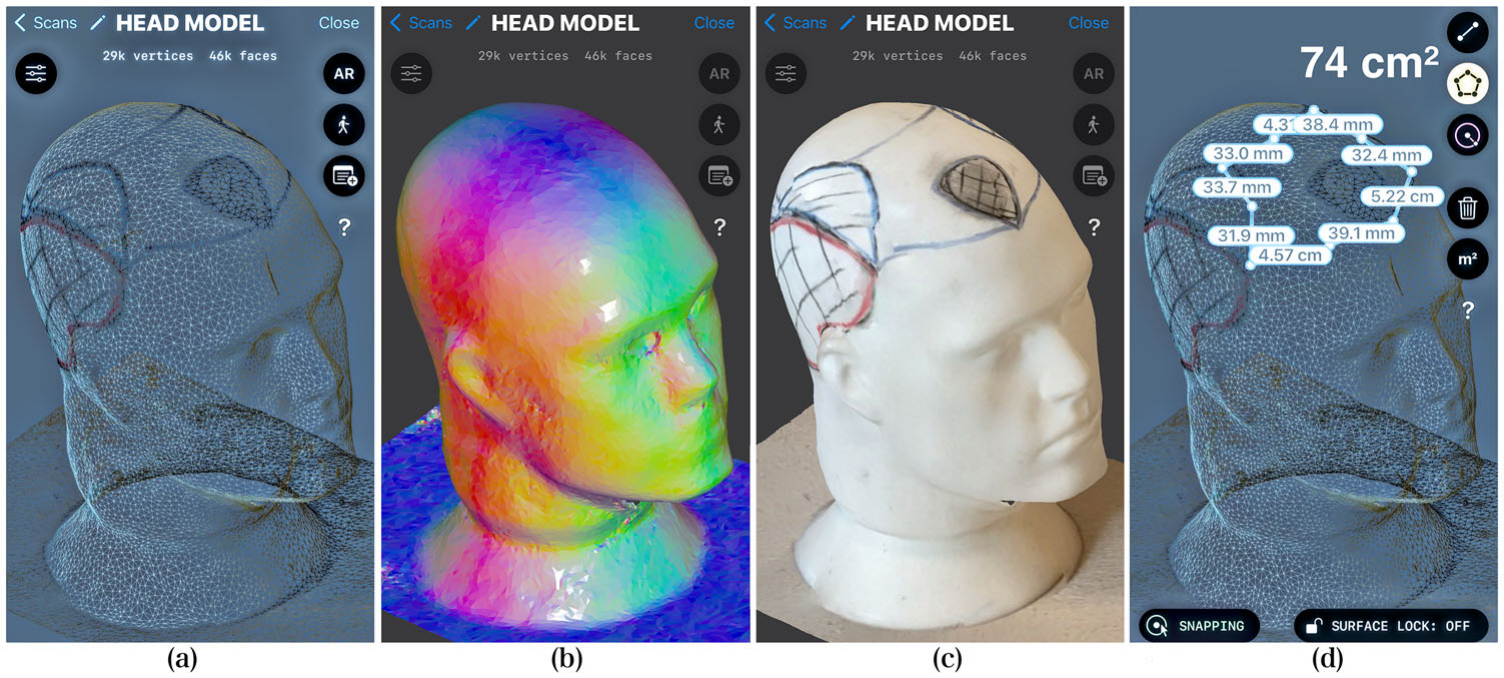
Example of measurement of surface area from 3D objects using LiDAR technology. **a** Wireframe; **b** Normals; **c** Rendered tri-dimensional model; **d** Surface area measurement. The dummy head 3D model was created using LiDAR technology contained in a mobile phone

First it creates a wireframe which consists of lines and shapes that define the edges and boundaries of different elements in the head surface (Fig. 4a). Then software tools calculate the normal vectors, which represent the orientation of the surface at each point (Fig. 4b). After that, a more detailed mesh model of the head is created, based on the LiDAR data, wireframe, and normals and then, finally the final 3D model is rendered, and can be viewed and analyzed from different angles and perspectives (Fig. 4c). As the distances are valued over the 3D mesh, the area is calculated on the surface of the head, thus representing a very accurate measurement of the hairless areas (Fig. 4d).

A similar technology called TrueDepth present on certain Apple devices can also be used to create a 3D model of the scalp over a very accurate 3D mesh. TrueDepth utilizes a combination of hardware components, including an infrared camera, flood illuminator, and dot projector, to capture depth information and generate a detailed 3D representation of the patient's scalp. The technology projects a grid of infrared dots onto the scalp, and the infrared camera captures the pattern by measuring the distortion of the dots. Using the depth data, TrueDepth technology can construct a 3D mesh of the user's scalp.

Before capturing the 3D model, it is important to wet the patient's hair and comb it back, so that all the bald and miniaturized areas can be easily identified for proper measurement.

#### Measurement by Photos

**Fig. 5:**
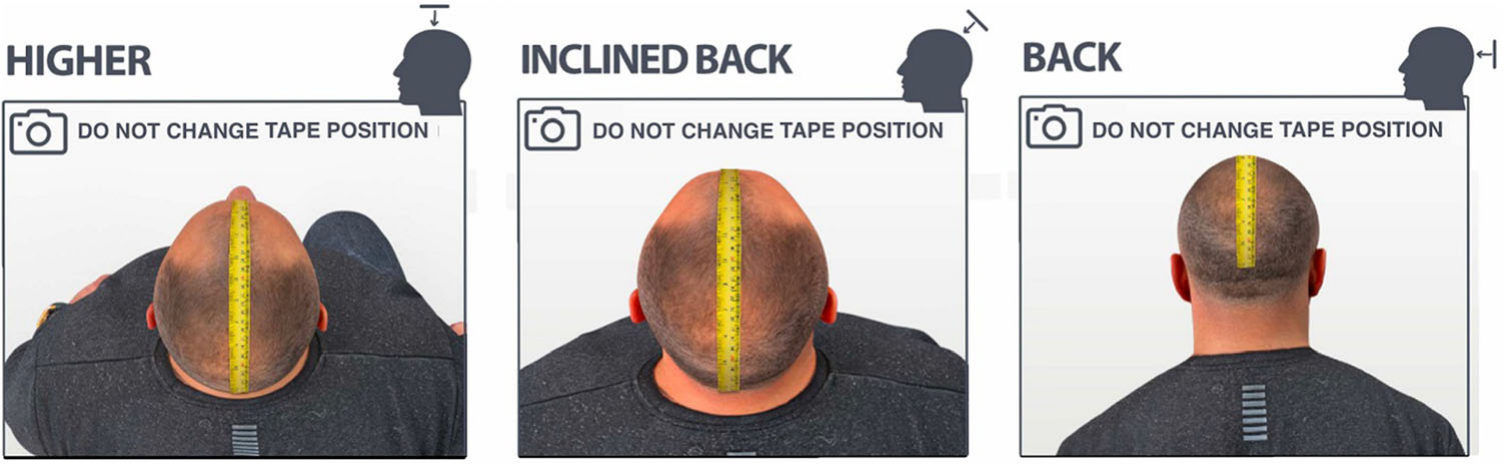
Positions to take the images for area measurement, using a measurement tape (yellow) as size reference. Images were created with CGI rendered 3D models in Adobe® Photoshop 2023 version 24.1.1

Photos are taken in the positions shown below using a size reference, preferably a measuring tape. The tape should be kept in the same position in all photos to mitigate the possibility of intersections between the regions, which are measured separately. With a still tape, clear limits can be established in the transition between the areas, for their subsequent summation. Using the humid hair combed back, photographs should be obtained in the following incidences (Fig. 5).

- Superior view (with the patient frowning to identify the hairline position): to measure the front and mid-scalp areas;
- Vertex view: to measure the crown area;
- Back view: to measure the area of the caudal extent of baldness in its most advanced classes.

**Fig. 6:**
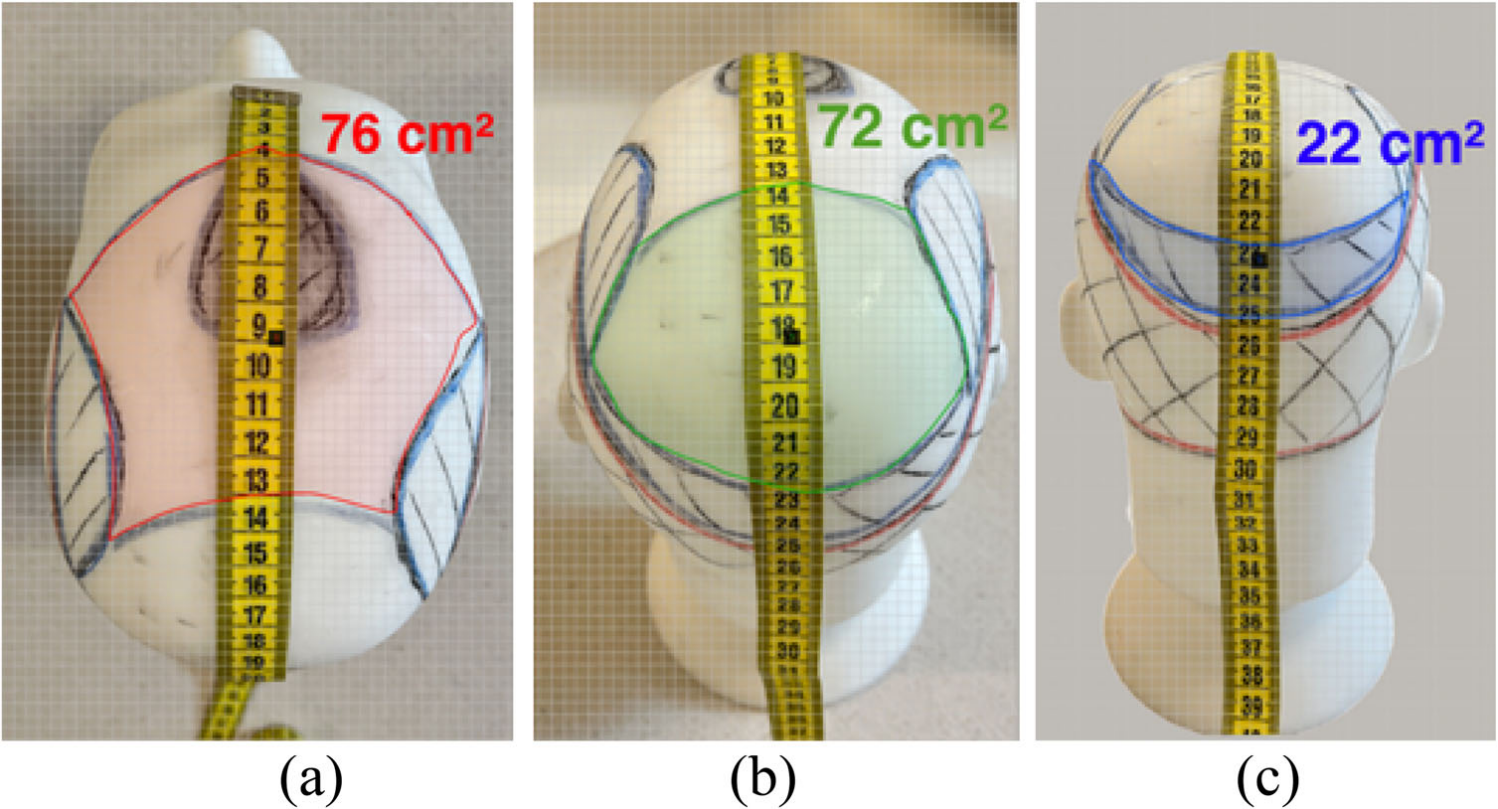
Measurement of hairless areas in photographs from different positions. **a** Measurement of frontal and mid scalp areas; **b** Measurement of Vertex area; **c** Measurement of occipital thinned area

The objective of the incidences specified above is to capture the hairless areas more accurately in the two-dimensional plane so that distortions determined by the cranial curvature are attenuated. The 3 photos are exported to a program or an app of area measurement; for each photo the specified region is measured and then they are summed up for the total bald area (Fig. 6).

#### Measurement by Square Centimeter’s Stamp

**Fig. 7:**
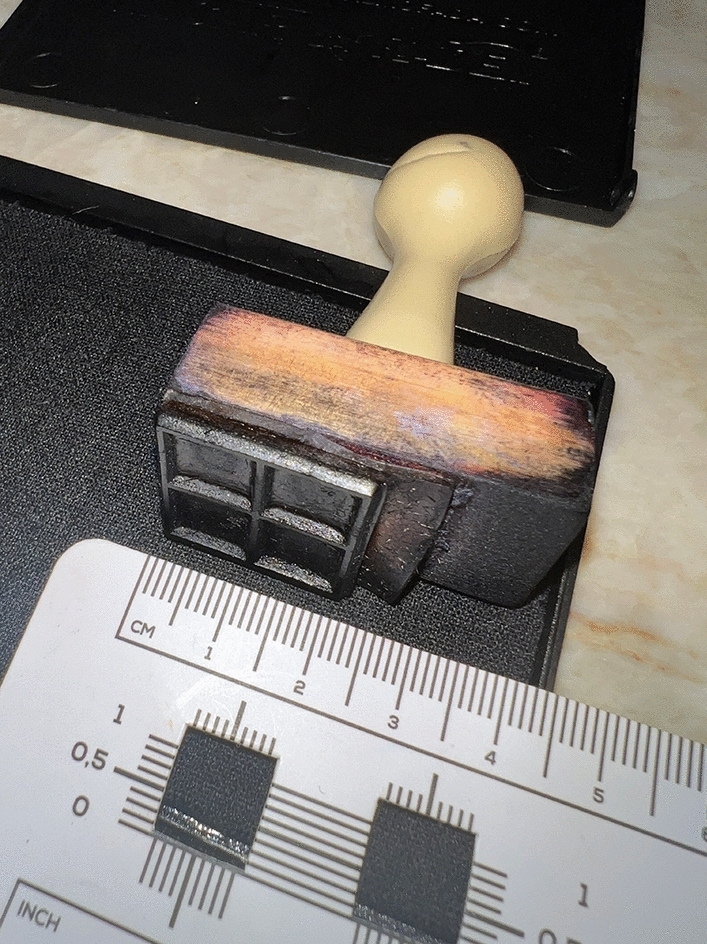
Square centimeter’s stamp. Author’s own equipment image

For the measurement of the bald area, the shaved head already marked for the HT must be stamped with a 4 cm^2^ gridded stamp divided into 1 cm^2^ (Fig. 7). The squares inside the pre-marked zone are counted and a very accurate extent is measured. The accuracy of this method may be similar to LiDAR’s. However, it is almost impractical on an unshaved head. Hence, it is recommended for conference and planning purposes in the immediate preoperative.

#### Measurement with Transparent Gridded Sheets

Transparent plastic sheets are marked with 1 cm^2^ grids using a permanent marker pen. The gridded sheet is placed over the desired scalp surface whose size is to be calculated and using a removable marker pen the bald and thinning regions are delimited and the squares are counted [19]. The removable ink is wiped, and the sheet can be used for other measurements.

#### Measurement with Ruler or Tape

The areas are roughly calculated using a tape or ruler. The examiner must divide the balding territory into polygons and calculate the areas of these polygons sequentially.

Each of the methods presented here has advantages and disadvantages that are listed in Table 3.

**Table 3:** Advantages and disadvantages of the methods of measuring the balding regions.

Method of measurement	Advantages	Disadvantages
3D mesh	Accurate, can be used in any kind of cranial surface, possible to measure areas with hair, does not require a size reference in the scene	Requires a specific device, demands training to use it, and takes time to process and render the 3D model
Photo	Moderately accurate, fast to calculate areas, doesn´t require special equipment, possible to measure areas with hair	Accuracy can be affected by cranial curvature; a misplaced size reference will impact directly in the calculated area
Square centimeter’s Stamp	Accurate; easy to use; accuracy almost unaffected by cranial curvature; does not require any special equipment	Can be performed only in a shaved head or on bald areas
Transparent Gridded Sheets	Cheap and reusable; easy to use; does not require any special equipment	Low-to-moderate accuracy as the cranial curvature greatly affects the positioning of the sheet
Ruler or tape	Low-cost procedure	Inaccurate

The limitations to measure the RBA may be related to distortions in the calculation of the area due to the difficulty of bringing a three-dimensional reality into the bidimensional plane. Especially for a virtual evaluation, photos taken by the patient are often unsuited for an accurate classification. If they are too close or taken in wrong angles or with the reference placed in a different plane from the bald area, they are unreliable for measuring the areas. In this case, the HMI must be determined without trichoscopy and this may be a problem for inexperienced surgeons as it may lead to underrated PRECISE classes. Specific guidelines to help young surgeons in hair transplantation techniques may be found in the literature [20].

As for the mesh measurement it demands training to obtain the perfect 3D model and requires compatible mobile phone, tablet or a specific device. Nevertheless, it is necessary to confirm the areas measuring them again in the immediate preoperative with the use of the stamp when the patient’s head is shaved. This will allow a final refinement of the strategy.

Although the scale may guide to a total number of grafts needed for a complete hair restoration in the assessed patient, the planning on how to achieve this number of grafts is up to the hair restoration surgeon. In other words, if the surgeon is facing a PRECISE class 5 he would need 7500 grafts to completely restore it. This implies in indicating 2 or 3 procedures for reaching this amount of FUs. But ultimately, the numbers of FU to be transplanted may vary slightly depending on the available resources and other nuances inherent to the patient's nature.

Of utmost importance, surgeons must also consider whether the patient has the resources (i.e. availability of hair follicles in donor areas) to achieve an ideal restoration. If the surgeon is facing a very advanced PRECISE class, he needs to consider adapting the surgical strategy to fit into the resources available, as for a complete hair restoration he would need a very large number of grafts that may not be possible to harvest in the patient donor areas. For instance, a PRECISE class 10 patient would need 15,000 grafts for a complete restoration of all the bald areas. This is such a number of grafts that is not achievable in a lot of patients, even using body hair transplant. In such situations, the surgeon should manage the patient's expectations into realistic perspectives and explain that the final achievable result may differ from what is ideal to him. This adaptation in surgical planning may include a higher hairline, no temporal restoration and intentionally transplanting low density on the crown area, for example. Consequently, marking a high hairline on a PRECISE class 10 may save 3000 grafts, or even more, from being transplanted. Therefore, a class 10 could be restored with around 12,000 grafts if the correct strategy is used.

The definition of strategies like the one mentioned above is also possible to be achieved through telehealth. This is due to the fact that the surgeon is able to quantitatively classify the baldness of a patient assisted by telemedicine using photo measurement programs in the PRECISE scale. This is a major advantage of the quantitative scale and will reduce the error brought by qualitative scales in the estimated number of FUs necessary in the procedure. For illustrative purposes, a Norwood-Hamilton class 3 patient which was classified as class 2.4 in the PRECISE scale would need around 3600 FUs in his procedure, by contrast, a Norwood-Hamilton class 3 patient classified as a PRECISE class 3.5 would need around 5250 FUs for a good result. Thereby, the PRECISE scale would also represent a rationale for adequate charging for grafts transplanted.

There are great divergences between examiners assessing the same patient and classifying him on the qualitative scales, showing that those scales are not reliable for creating a surgical plan. Differently, due to its numerical nature, the PRECISE scale should be endowed with good reproducibility, i.e., two examiners should reach the same class for the same patient. On that ground, it is expected that the routine application of the scale can speed up the learning curve for beginners in the field of hair restoration surgery.

For the near future, we aim to study and validate in greater detail the variation in the classification among users of the scale, in order to better understand its performance in terms of reproducibility. It would also be important to verify how the scale correlates with the coverage value (HCV) and the hair diameter index (HDI) for a possible subdivision of the surgical strategy according to the different features of the hair of each patient and the regions that will receive the grafts. Finally, also relevant will be the analysis of the impact in the learning curve of beginners in the hair restoration field. Ultimately, through the development of artificial intelligence, the improvement of tools may minimize errors in area measurements and facilitate the calculation of the HMI, while the scale can have its performance maximized.

## Conclusion

The PRECISE scale was developed with the main purpose of classifying AGA quantitatively, for an accurate planning of hair transplantation procedure. Therefore, the scale was able to accurately define MPHL quantitatively in the simulations presented. It was able to reduce distortions of qualitative scales, and to explain cases in which greater amounts of FUs are needed to cover a relatively lower class of baldness. In addition, it is expected to be reproducible among different examiners as it does not rely on qualitative features.
